# Assessing the level of radiation experienced by anesthesiologists during transfemoral Transcatheter Aortic Valve Implantation and protection by a lead cap

**DOI:** 10.1371/journal.pone.0210872

**Published:** 2019-01-30

**Authors:** N. Patrick Mayr, Gunther Wiesner, Angela Kretschmer, Johannes Brönner, Herbert Hoedlmoser, Oliver Husser, Albert M. Kasel, Rüdiger Lange, Peter Tassani-Prell

**Affiliations:** 1 Institut für Anästhesiologie, Deutsches Herzzentrum München, Technische Universität München, Munich, Germany; 2 Deutsches Herzzentrum München, Technische Universität München, Munich, Germany; 3 Helmholtz Zentrum München, German Research Center for Environmental Health, Munich, Germany; 4 Klinik für Herz- und Kreislauferkrankungen, Deutsches Herzzentrum München, Technische Universität München, Munich, Germany; 5 Klinik für Herz- und Gefäßchirurgie, Deutsches Herzzentrum München, Technische Universität München, Munich, Germany; University of Mississippi Medical Center, UNITED STATES

## Abstract

**Objective:**

Transfemoral Transcatheter Aortic Valve Implantation (TAVI) has become a standard therapy for patients with aortic valve stenosis. Fluoroscopic imaging is essential for TAVI with the anesthesiologist’s workplace close to patient’s head side. While the use of lead-caps has been shown to be useful for interventional cardiologists, data are lacking for anesthesiologists.

**Methods:**

A protective cap with a 0.35 lead-equivalent was worn on 15 working days by one anesthesiologist. Six detectors (three outside, three inside) were analyzed to determine the reduction of radiation. Literature search was conducted between April and October 2018.

**Results:**

In the observational period, 32 TAVI procedures were conducted. A maximum radiation dose of 0.55 mSv was detected by the dosimeters at the outside of the cap. The dosimeters inside the cap, in contrast, displayed a constant radiation dose of 0.08 mSv.

**Conclusion:**

The anesthesiologist’s head is exposed to significant radiation during TAVI and it can be protected by wearing a lead-cap.

## Introduction

To date about 350.000 transfemoral Transcatheter Aortic Valve Implantations (TAVI) have been performed [1] with more than 13.000 in Germany in 2015 alone [2]. As periprocedural fluoroscopic imaging is essential, this procedure is done in cardiologic catheterization laboratories (CathLabs) [3] or specially designed Hybrid-ORs. As several steps of the procedure, may be painful to the patient or haemodynamically impairing, anesthesia managed care is mandatory for these patients. Thereby, the anesthesiologist’s workplace is close to the patient’s head side. Periprocedural pain and unrest, or cases of emergency may demand a close contact of the anesthesiologist to the patient. Furthermore, the X-ray protection, attached to the CathLab-table, is often deficient at the head-side and a head-sided position of the C-arm may further impede stationary X-ray protection. In recent years, radiation protection has become an important topic for the interventional cardiologists [4] and reports of an increased incidence of left sided brain tumors have been published [5, 6]. It has been shown that the use of lead caps and glasses could reduce the radiation exposure of the brain in this group [7–9]. Further data suggest that the occupational radiation exposure of the medical staff during TAVI is highly dependent on the access site used (e.g. transapical vs. transfemoral), protection shields, angulation of the C-arm and beam projection [10–13]. Such data is lacking for the anesthesiologist positioned at the head-side of the patient during TAVI. Therefore, this study was designed as a pilot and it was hypothesized that, despite the presence of a protective shield, the head of the anesthesiologist is exposed to radiation during TAVI procedures. Furthermore, it was investigated whether the use of a lead cap could reduce the radiation exposure of the head.

## Methods

This trial was a single-center observational pilot-study performed at the Institut für Anästhesiologie, Deutsches Herzzentrum München, a university-hospital specialized in cardiovascular diseases, between February and March 2016. The decision for TAVI and the type of transcatheter valve was at the discretion of the institution´s heart team. All cases were conducted in the same catheterization laboratory using a Philips ALLURA XPER FD 10 X-ray system (Philips Medical Systems Nederland B.V., Best, Nederlands). Informed consent and ethical approval was waived by the ethical committee of the Technische Universität München, as the measurements were done during routine procedures.

### Endpoints

The reduction of the radiation exposure at the head of the attending anesthesiologist by the use of a lead head cap was chosen as primary endpoint.

As a secondary endpoint, the exposure of the anesthesiologist´s head to radiation during these procedures was assessed. “Exposure” was defined as the maximum radiation to which the head would have been exposed, if no lead cap would have been worn.

**Fig 1:**
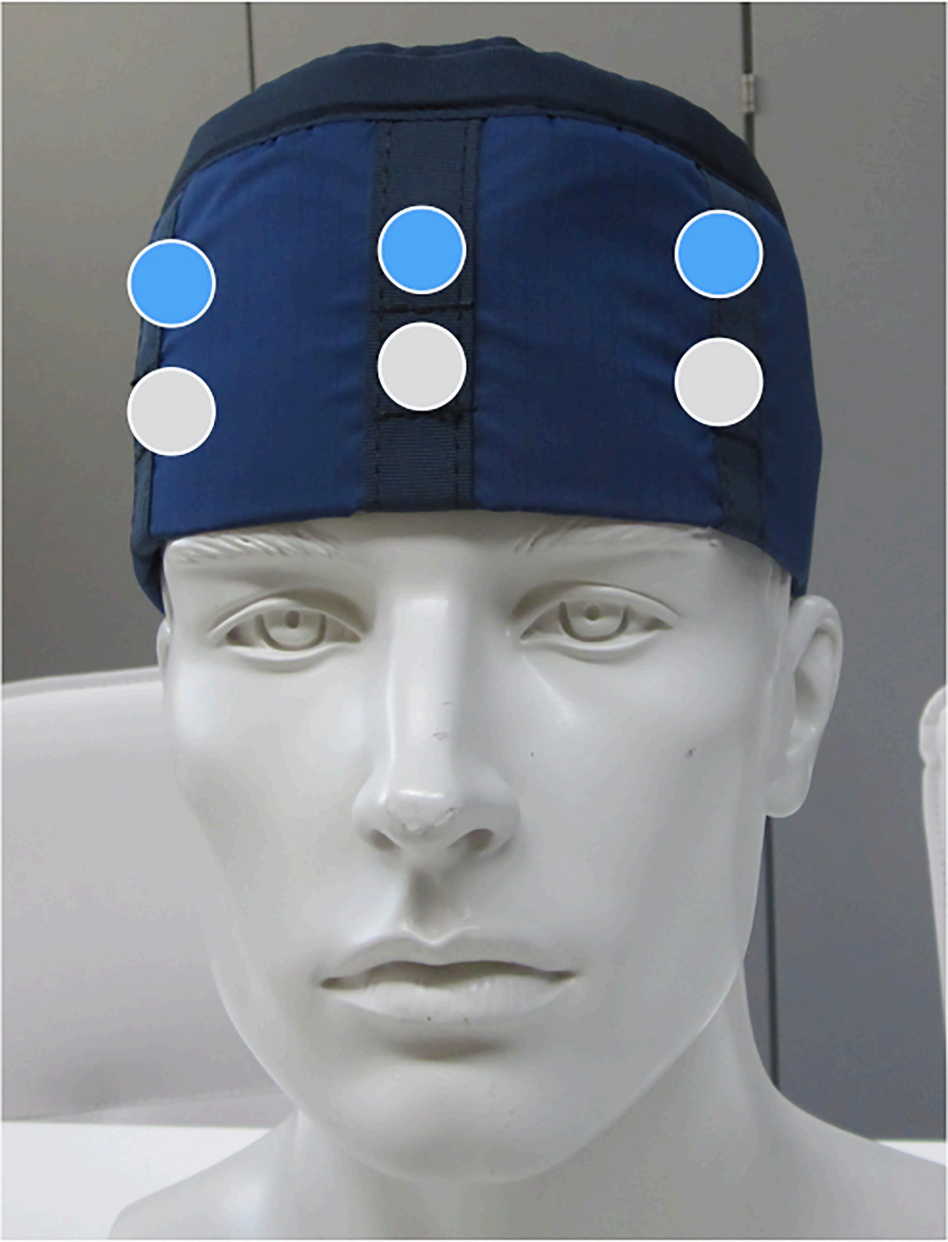
Position of the detectors outside (grey) and inside (blue) the cap.

A personalized cap consisting of Novalite (Mavig, Munich Germany)–a lead composite material with a 0.35 mm lead equivalent–was used. To ensure comparable conditions, i.e. reproducibility of the results, the same cardiac anesthesiologist utilized the cap over a period of 15 work-days performing TAVI procedures. Six detectors—three at the outside of the cap and three at the inside (left side, right side and in the center of the forehead)—were analyzed, as shown in Fig 1.

### Anesthesiologist’s standard radiation protection

**Fig 2:**
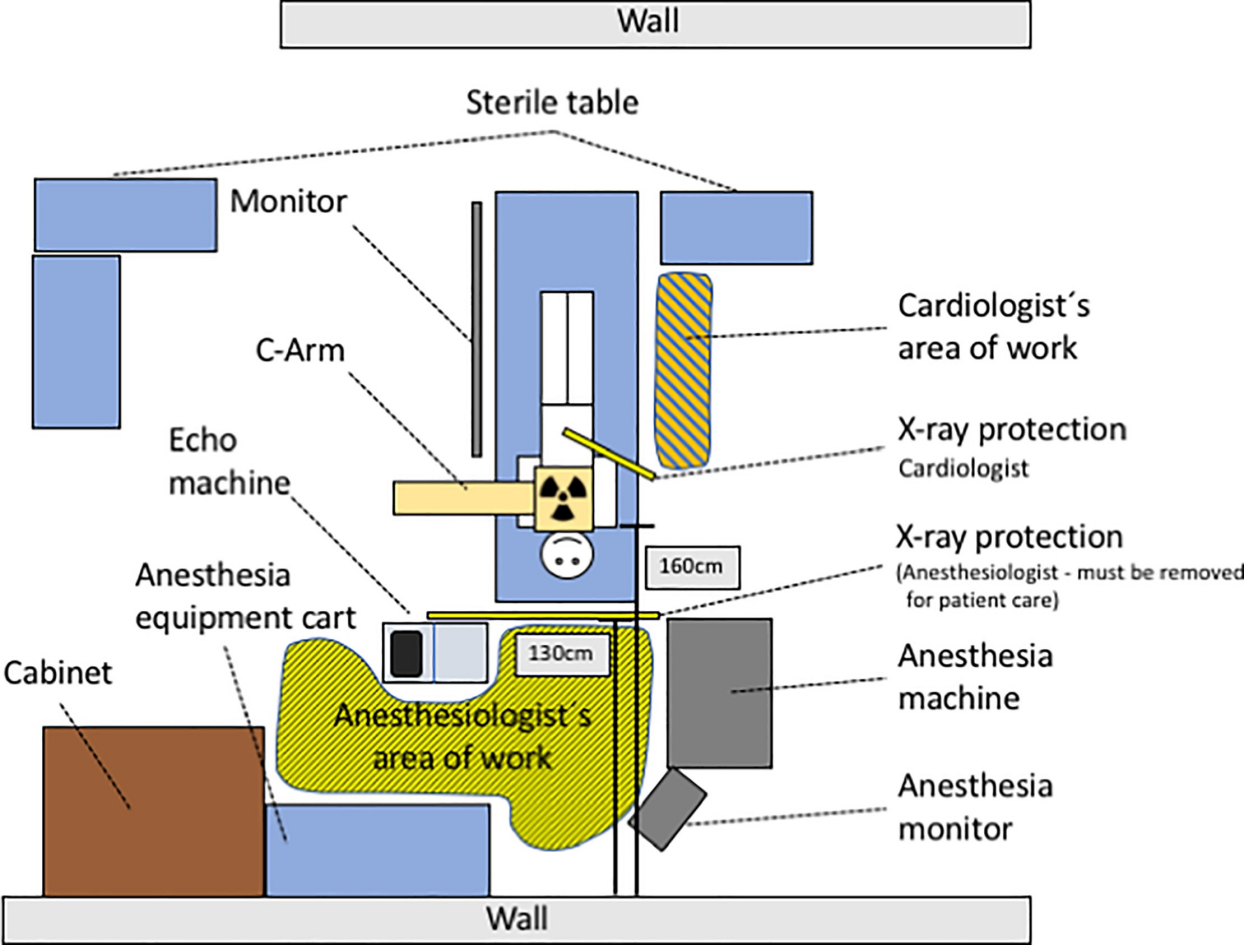
Room setup in the CathLab.

The anesthesiologist’s personal protection consisted of a personalized lead coat (including upper arm cover), radiation protection glasses and thyroid protection. In accordance to German Federal Regulations, a personal dosimeter was worn at chest height underneath the protective coat. The position of the anesthesia machine and monitoring, as well as the position of the protection shield is shown in Fig 2. The top level of the lead shield was at 145 cm, while the anesthesiologist’s height was 182 cm, therefore the head of the anesthesiologist is actually beyond the mobile radiation shield.

As imaging modes, fluoroscopy and cine-angiography were used during the procedure. A low-dose program and commonly applied measures for radiation protection as focusing on the area of interest and minimizing the distance between patient and flat-panel detector were used [14]. Demographic data of all patients were recorded and the operative risk was assessed by the EuroSCORE II [15].

Procedure time, radiation time and dose area product (DAP) data were collected for each patient. DAP was used as a surrogate parameter for the total amount of radiation energy and therefore also as a relative indicator for the scatter dose the anesthesiologist is exposed to.

Procedures were conducted in general anesthesia or conscious sedation based on the patient´s comorbidities. Both anesthesia techniques have been described in detail before [16].

### Measurement

Due to the spatial constraints inside the cap small detectors were required for all measurements. At the Individual Monitoring Service of the Helmholtz Zentrum München (HMGU) different dosimeters are used for the measurement of the operational radiation protection quantities H_p_(10), H_p_(3) and H_p_(0.07) which provide conservative approximations for the effective dose, eye lens dose and skin dose, respectively [17–19]. All dosimeters consist of a passive detector and a dosimeter enclosure or holder. Due to the limited space inside the lead cap these enclosures could not be used in the experiment. The measurements were performed with LiF thermoluminescence detectors (specifically LiF:Mg,Cu,P material, i.e. MCP-N material), which are also utilized in extremity dosimeters like finger ring dosimeters. These detectors were adequate for the special requirements regarding detector size and high sensitivity.

It is imperative to carefully address the issues evaluating detector signals that may be biased by the omission of the dosimeter enclosures and the nonstandard placement attached to a lead cap. The calibration of a dosimeter as used in the automated analysis systems at the HMGU assumes correct placement of the dosimeter on a dedicated position on the body and considers backscattered radiation from the body as well as radiative processes inside the dosimeter enclosure such as shielding of low energy radiation and signal build up with higher photon energies. The use of the lead cap itself modifies the radiation environment by causing different backscatter for the detectors on the outside and hardening of the energy spectrum propagating to the inside of the cap. Therefore the result of such a dose measurement cannot be interpreted as a correct value for Hp(3) or Hp(0.07) in the strict metrological sense. However, the measurements can still be used to reliably estimate the effectiveness of the shielding of the cap by calculating the relative signal between the detectors outside and inside of the cap and by estimating the uncertainty of this relative measurement due to the modified photon spectrum and differences in backscatter.

### Data evaluation

The cumulative radiation dose outside and inside the lead cap was measured and the ratio inside to outside was calculated.

## Results

During the observational period a total of 32 transfemoral TAVI procedures was conducted in the cardiologic CathLab within 15 working days. Patient´s baseline and procedural data are shown in Table 1.

**Table 1:** Patient´s characteristics.

Patient and procedural characteristics	
**Male patients (n = )**	20/32 (63%)
**Age (years)**	80 (75.5–84)
**Body Mass Index**	22.4 (20.3–24.9)
**EuroSCORE II (%)**	3.41 (2.28–6.23)
**Balloon-expanding valve (n = )**	24 (75%)
**General anesthesia (n = )**	16 (50%)
**Operation time (Min)**	63 (50–75)
**Radiation time (Min)**	11.4 (9.5–14.9)
**Dose Area Product (DAP) (μGym**^**2**^**)**	752 (382–1729)

Quantitative data are given in absolute numbers and percentage. Continuous data are given as Median (Interquartile Range)

All procedures were conducted without procedural or anesthesiologic complications. No unplanned conversions to general anesthesia or major vascular injuries were registered.

**Fig 3:**
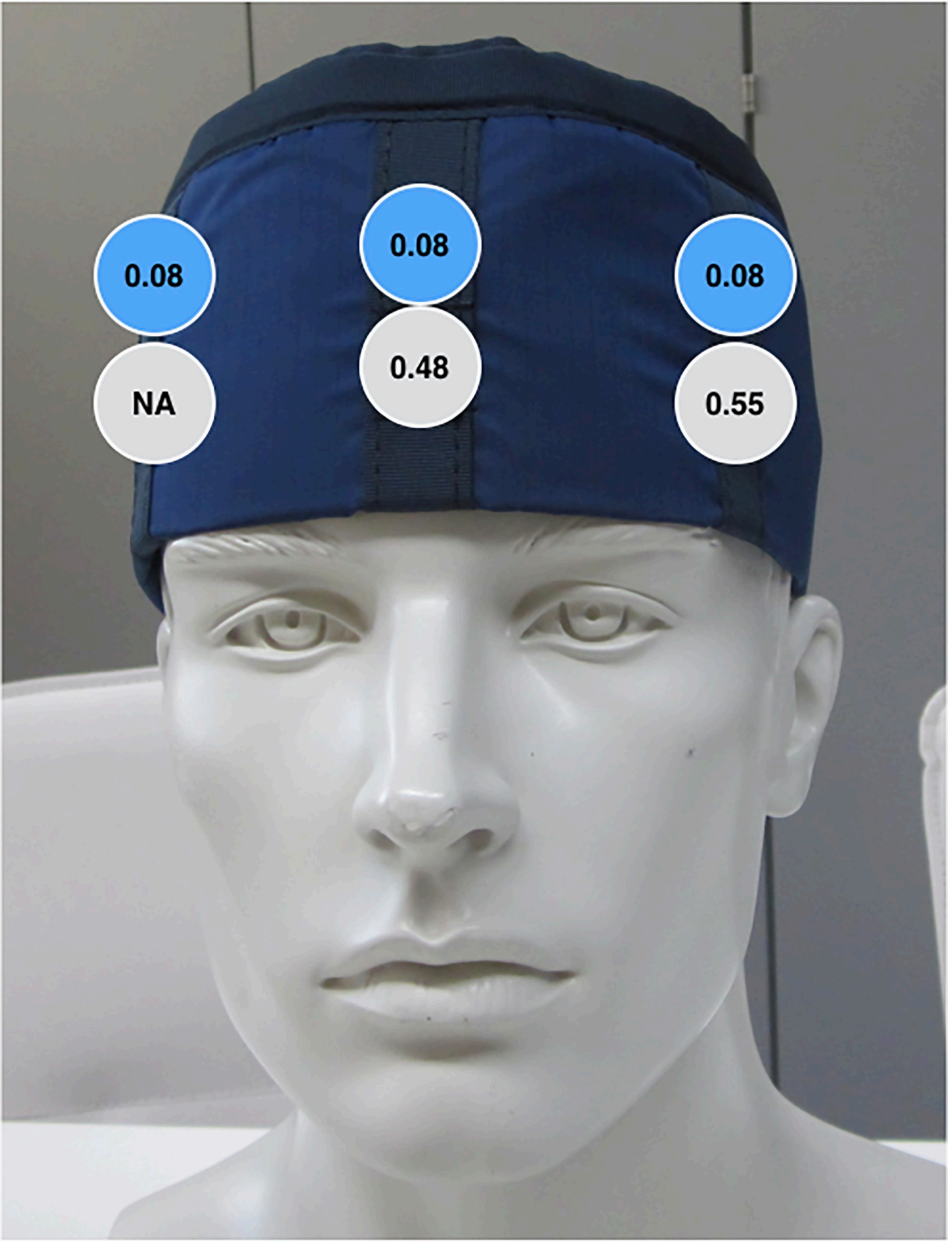
Radiation measurement inside and outside the cap in mSv Hp(3) (relative uncertainty ~35%, grey–outside; blue - inside): The relative uncertainty is defined as the expanded uncertainty (k = 2) divided by the measured dose value.

The right outside dosimeter was discovered defect after measurements and therefore excluded from analysis. As shown in Fig 3 the highest radiation dose was detected on the left outside of the cap. Inside, there were comparable measurements at all three positions (left, right, center). Compared to the outside measurements, the radiation dose inside the cap was reduced as shown in Table 2.

**Table 2:** Radiation measurement outside and inside the cap in mSv Hp(3).

Detector position	Value outside	Value inside	Relation to the corresponding outside position	Relation to the point of highest radiation exposure
**Left**	0.55 mSv Hp(3)	0.08 mSv Hp(3)	15%	Outside: reference Inside: 15%
**Middle**	0.48 mSv Hp(3)	0.08 mSv Hp(3)	17%	Outside: 87% Inside: 15%
**Right**	NA	0.08 mSv Hp(3)	NA	Outside: NA Inside: 15%

Therefore, the anesthesiologist´s head would have been exposed to a maximum of 0.55 mSv, if no lead cap would have been worn.

## Discussion

Despite the presence of a radiation protection shield, the anesthesiologist´s head was exposed to radiation. This is due to the fact that the top height of the protective shield was at 145 cm while the anesthesiologist was 182 cm in height Furthermore, it could be shown for the first time in this real-life setting that the use of a particular protective cap facilitates a significant reduction of radiation, the acting anesthesiologist may be exposed to. Different other radiation protection caps are available, partially also covering the oral mucosa and parotis glands. All clinically evaluated models have shown the ability to reduce the radiation exposure significantly between 62% and 90% [7, 8, 20, 21] in interventional cardiologists. Our measurements showed a reduction of 83–85%. Therefore, the protective potential for the anesthesiologist appears to be comparable with those for interventional cardiologists.

For many decades the brain, as a highly differentiated organ, has been considered to be radio-resistant according to the “Law of Bergoiné and Tribondeau” [22]. Newer research is contradictionary to this point of view [23] and an increasing number of interventional cardiologists with left sided brain tumors seem to support the thesis of a causal relationship [6]. Vascular and neurocognitive effects of radiation exposure have also been described [23, 24], but the occupational significance is still unclear at the moment. The head´s radiation exposure has shown to be related to the position of the physician with a focus on the left side [25]. As it can be expected from the cardiologist’s area of work (Fig 2), radiation protective caps have been proposed to be useful in addition to lead-glass shields [20, 25] and protective glasses [26]. Radiation exposure of the anesthesiologist working at the head of the patient is dependent on different variable factors like the angulation of the C-Arm, the dosis-area-product, volume-dependent scatter radiation and dose acquisition setting [27]. As several parts of the body (e.g. the face, lower legs, arms) are still uncovered, a multimodal approach to reduce the radiation exposure [14] is necessary. This includes the limit of radiation usage, decreased cine use, a detector positioned close to the patient, a decreased frame rate, software magnification and real-time dose monitoring [27–29]. Besides these techniques personalized (and fitting) radiation protection equipment (apron, thyroid shield, glasses) represent the current state of the art and have shown to reduce the exposition of the covered areas significantly [12]. Currently, only a dosimeter (worn at the chest below the lead coat) is mandatory in Germany.

For all heart valves, interventional procedures for the treatment of acquired structural heart disease are now available with increasing numbers. Anesthesia care for a considerable number of these patients with a multitude of comorbidities is mandatory. As a consequence, working in radiation environment like the CathLab will become more and more for anesthesiologists. Although radiation dosage has decreased by implementation of new technologies [30] and procedural experience [31] worldwide, the anesthesiologist’s exposure to radiation is generally considered to be higher in TAVI than in other interventional procedures [12, 32]. Newer protection systems for interventional cardiologists [12] are not suitable for anesthesiologists as they are designed for a static position and not for variable working positions. A solid shield may protect the anesthesiologist during the routine uneventful procedure. With 145 cm of height and 81cm width, the lead shield was lower than the head of the anesthesiologist. With a greater shield height, the access to the patient´s head would be impaired. Same is true for a hanging protective shield. Such a shield might protect the head of the anesthesiologist during a static position, but such a broad shield impairs the access to the patient’s head or the positioning of the C-Arm from the head side.

But in the presence of severe adverse events, demanding a mask ventilation or emergency induction of general anesthesia, this protection shield has to be removed. Despite a ten-year experience, severe adverse events still occur [33] in a considerable number of patients. A recent analysis of transfemoral TAVI procedures revealed a rate of conversion from sedation to general anesthesia in nearly 6%. Most of these conversions were due to procedural complications, followed by the need for general anesthesia due to patient´s pain or discomfort. While for the latter, the procedure–and fluoroscopy–can be interrupted, this might not be possible during procedure related complications. Aortic annular rupture, pericardial effusion or hemodynamic compromising aortic regurgitation require an immediate diagnosis and therapy. This must usually be done under fluoroscopy, with the anesthesiologist´s head close to the radiation device without protection. Therefore, it can be assumed that the exposition to radiation is markedly increased in these situations.

In 32 uneventful TAVI-procedures, a maximum outside radiation of 0.55±0.2 mSv was detected which translates into ~0.69±0.23 mSv Hp(3) with a backscatter correction applied to account for the presence of the lead cap. The exposure was highest at the left frontal side. This is caused by the working position of the anesthesiologist. In our setting, the anesthesia-equipment and monitoring are positioned at the right head side of the patient. As a consequence of this setting, the anesthesiologist has to work with the left frontal head side towards the radiation.

## Limitations

The data obtained in this trial apply only for this specific equipment and setting and might not be generally applied. One limitation of this study is that the measured dose values in terms of H_p_(3) exhibit somewhat large uncertainties due to the use of non-enclosed detectors and their placement inside and outside the lead cap. The uncertainties of the measurements were estimated by means of data from the uncertainty analysis of the HMGU extremity dosimeters [34] and by means of additional calibration measurements with the detectors and the lead cap on the ISO cylinder phantom [35] (head phantom) at the calibration facilities of the HMGU. All uncertainties in the following are given as expanded uncertainties [36] with a coverage factor of k = 2, i.e the 95% confidentiality level. The largest dose value outside the lead cap was 0.55±0.2 mSv, with the dominating uncertainty contributions arising from the energy response of the detector and statistical uncertainty. However, in the calibration measurements with the lead cap and a head phantom it was shown, that the presence of the lead cap shields the detector from backscattered radiation from the head phantom, therefore the actual value of the eye lens dose for a person not wearing a lead cap would be about 15%-25% higher depending on the radiation quality. The measured values inside the lead cap are in the order of 0.08±0.03 mSv. In this case the detector is used near the detection limit, which increases the uncertainties mostly due to background subtraction, however this effect could be alleviated by means of dedicated background dosimeters. Even though the measurement uncertainties are pronounced at these small dose values, the relative measurement between the outside and inside levels, i.e. the determined decrease of 85% exhibits a comparatively smaller uncertainty of approximately 11% due to the laws of error propagation. Therefore, the conclusion of the effectiveness of the shielding capability of the lead cap is valid.

Currently, about 650 transfemoral TAVI procedures per year are performed in our institution. Considering that this number increases and that the course of about 9% [37] is not uneventful and accompanied by a higher radiation exposure, it would be prudent for anesthesiologist to wear a lead cap to complete his personal protective equipment.

## Conclusion

In this pilot study, we hypotized that the head of the anesthesiologist is exposed to radiation during TAVI. Furthermore, we evaluated the use of a lead cap to reduce the radiation exposure. In 32 uneventful TAVI procedures, the head was exposed to significant radiation. This primary due to the fact, that the protective shield was lower than the head of the anesthesiologist during routine care. As the anesthesia equipment and monitoring was placed on the right head side of the patient, the radiation exposure of the anesthesiologist´s head was highest on the left side. By wearing the lead cap during these procedures, a reduction of the heads radiation exposure of about 85% was achieved. Despite these findings, structural radiation protection, like protection shields, still remains of high importance.
